# The short-term outcomes of cementless stem for hip arthroplasty in the elderly patients: comparison with patients < 65 years

**DOI:** 10.1186/s12891-022-06025-x

**Published:** 2022-12-07

**Authors:** Jun Wang, Zhibo Deng, Bin Huang, ZhengMing Zhao, HaiMing Wan, Hong Ding

**Affiliations:** 1 Department of Orthopaedic Surgery, the First People’s Hospital of Chongqing Liangjiang New Area, Chongqing, 400010 China; 2 Department of Orthopedics, Shengli Clinical Medical College of Fujian Medical University, Fujian Provincial Hospital, 350001 Fuzhou, China

**Keywords:** Cementless stem, Elderly, Hip arthroplasty, Sinking distance

## Abstract

**Objective:**

The use of cement or cementless femoral stem prosthesis for hip arthroplasty in elderly patients has been controversial. This study investigated the efficacy and safety of cementless femoral stem in elderly patients (age ≥ 65 years).

**Methods:**

The short-term efficacy of primary hip arthroplasty with cementless femoral stem in our hospital from January 2014 to June 2021 was retrospectively analyzed. Patients were divided into two groups according to age: young group (< 65 years) and the elder group (≥ 65 years). The evaluation index was the Harris Hip score (Harris), postoperative femoral stem prosthesis sinking distance and femoral plant-related complications (periprosthetic fracture, prosthesis loosening, infection, dislocation, etc.).

**Results:**

There were 72 cases of 86 hips in the young group and 83 cases of 92 hips in the elder group. The recovery trend of functional score in the elder group was similar to that in the young group, and the Harris score increased from (38.35 ± 18.21) before surgery to (86.91 ± 12.55) at last follow-up (*p* < 0.01). Compared with the two groups at the same time of 3 months, 6 months and 12 months after operation, the sinking distance of the elder group was significantly greater (*P* < 0.05). Even in the elder group, there was a significant difference in sinking distance between 6 and 3 months after surgery (2.44 ± 0.49 mm vs 2.10 ± 0.65 mm, *P* = 0.004). However, there was no significant difference between 12 and 6 months (2.53 ± 0.53 mm vs 2.44 ± 0.49 mm, *P* = 0.40). A total of 10 patients in the elder group and 6 patients in the young group had a complication event (*P* = 0.36).

**Conclusions:**

Elderly patients with cementless femoral stems can achieve metal-bone integration slightly longer than young patients, but short-term clinical outcomes can be restored to a satisfactory level with adequate safety. However, the long-term efficacy still needs to be verified by multicenter, large sample size and prospective clinical follow-up results.

## Introduction

Hip arthroplasty (including total hip arthroplasty and hemiarthroplasty), as a mature surgical technique, has achieved good clinical results worldwide [1]. At present, in elderly patients undergoing primary hip replacement, femoral stem prosthesis is usually fixed with bone cement to obtain good follow-up results and long-term survival rate of prosthesis [2]. However, the use of Bone cement prosthesis may lead to the Bone cement implantation syndrome (BCIS) [3], which may lead to severe arrhythmia and cardiac arrest in severe cases. In the treatment of Bone cement hemiarthroplasty for femoral neck fracture, BCIS may carry up to a 16-fold increase in 30-day postoperative mortality [4]. Cementless femoral stem prosthesis can achieve immediate stability by closely embedding the medullary cavity of the proximal femur, and long-term stability of the prosthesis can be obtained by the host bone growing into the microporous layer on the surface of the prosthesis in the later stage [5]. For young patients and patients with high mobility requirements, long-term follow-up shows that cementless prosthesis can achieve higher hip score [6]. However, recent study believe that hip replacement with cementless prosthesis can also achieve good imaging and clinical results in elderly patients [7].

Tanzer et al. [8] found that cementless stem had a higher rate of revision in the early stage for patients older than 75 years(≤ 3 months) by comparing cement femoral stem, but no significant difference in complications after 3 months. In a network meta-analysis, Migliorini et al. [9] demonstrated that there were no significant differences in functional outcomes and complication rates between cement and cementless stem in elderly patients, but a tendency for lower mortality, revision and dislocation rates in cemented implants was evidenced. Gonzalez et al. [10] demonstrated that early revision for periprosthetic fracture was more prevalent in patients over the age of 65 years with cementless stem. Aaccording to the above literature, it has been controversial whether the femoral stem prosthesis should be fixed with cement or cementless in elderly patients. However, the current focus of studies [10, 11] comparing cement and cementless is still limited to older patients. Few studies have been conducted to compare the efficacy of cementless stem between the elderly and the young, especially in terms of radiological results.

Therefore, this study intends to compare the short-term follow-up results of cementless femoral prosthesis in different age groups by retrospective comparative study to observe the early functional score and prosthesis sinking of cementless femoral stem prosthesis in elderly patients, and to explore whether the cementless femoral stem fixation in elderly patients can achieve the same efficacy and safety as that in young patients.

## Materials and methods

### Patient selection

Patients who underwent hip arthroplasty with cementless femoral stem in our hospital from January 2014 to June 2021 were followed up. Inclusion criteria: ① Primary hip arthroplasty (including total hip arthroplasty and hemiarthroplasty); ② Surgeries were performed by the same doctor; ③ The femoral side is a cementless fixation prosthesis; Complete follow-up data (linical outcomes and radiographic data) were available at 1, 3, 6 months and 1 year after surgery. Exclusion criteria: ① Patients with other hip fractures other than the femoral neck; ② The patient was congenital hip dysplasia requiring osteotomy and reduction; ③ Patients with other serious medical diseases were followed up for less than 1 year; Patients with femoral pathological fracture or deep infection. It is worth noting that the retrieval process was completed by two scholars independently. Any inconsistency is resolved through discussion.

Finally, the patients were divided into elder group(≥ 65 years) and young group(< 65 years). The study was approved by the ethics committee of our hospital, and the requirement for informed consent was waived because it was a retrospective study. The accessed patient data complied with relevant protection, privacy guidelines and regulations.

### Surgical procedure

All operations were performed primarily by the same surgeon. The doctors used the conventional posterolateral approach of the hip joint, exposed the visual field of the hip joint operation area, cut off the femoral neck 1–1.5 cm above the lesser trochanter, and gently removed the femoral head. For patients undergoing total hip arthroplasty, an appropriate cementless acetabular cup was implanted by compression fitting. Next, preparation was made for the implantation of the femoral prosthesis. Then preparation was made for the implantation of the femoral prosthesis. The medullary cavity of the proximal femur was opened and expanded until the size was basically consistent with the model based on the preoperative plan, and whether the model prosthesis was stable in the medullary cavity was tested. A suitable size model of the femoral head is installed and the hip joint is repositioned. The assistant makes the hip joint do 120° forward flexion and 30° internal rotation to determine whether the artificial hip joint can be dislocated under normal movement. The femoral stem and femoral head prostheses were then replaced with standard biotype prostheses, and the incision was finally closed layer by layer. The femoral stem and femoral head prostheses were then replaced with standard biotype prostheses, and the incision was finally closed layer by layer. Enoxaparin 4100 IU was given subcutaneously 12 h after operation, once a day. Enoxaparin 4100 IU was given subcutaneously 12 h after operation, once a day. After discharge, rivaroxaban tablets were administered orally until 5 weeks postoperatively to prevent thrombosis. Within 24 h after surgery, the patient was instructed to move. Within 24 h after surgery, patients are encouraged to get out of bed and walk.

The stems included the Tri-Lock bone preserving stem (BPS) (Depuy, Warsaw, Indiana) or M/L Taper stems (Zimmer, Warsaw, IN). Both of the stems are made of titanium (Ti6Al4V alloy). The M/L Taper stem, which is a common type of cementless and collarless stem with a 12/14 taper design, has a single-wedge design with a proximal plasma coating, is tapered in the medial–lateral plane and is flatted in the anteroposterior plane. The Tri‐Lock BPS is a short tapered-wedge stem with a relatively short length and proximal GRIPTION microporous coating. Its design features include a highly polished and curved surface design at the distal end of the stem, distal flutes and a minimal lateral shoulder. Another feature is the maximum bone preserving.

### Outcome assessments

#### Clinical evaluation

The X-ray of hip joint was reexamined within 3 days after operation, and the follow-up was completed in the outpatient department at 1, 3, 6 months and 1 year after discharge, and then once a year. The Harris hip score (Harris) was used for hip function. The total score ≥ 90 was considered as excellent, 80–89 as good, 70–79 as medium, and < 70 as poor. Intraoperative and postoperative complications included thigh pain, heterotopic ossification, deep infection, dislocation, aseptic loosening, deep vein thrombosis, periprosthetic fractures, etc. As defined by Barrack et al. [12], pain was considered and recorded as thigh pain when it was on the anterior and/or lateral thigh below the inguinal area. Visual analog scale (VAS) score of 10 points was used, with 0 indicating no pain and 10 indicating severe pain.

#### Radiographic analysis

**Fig. 1:**
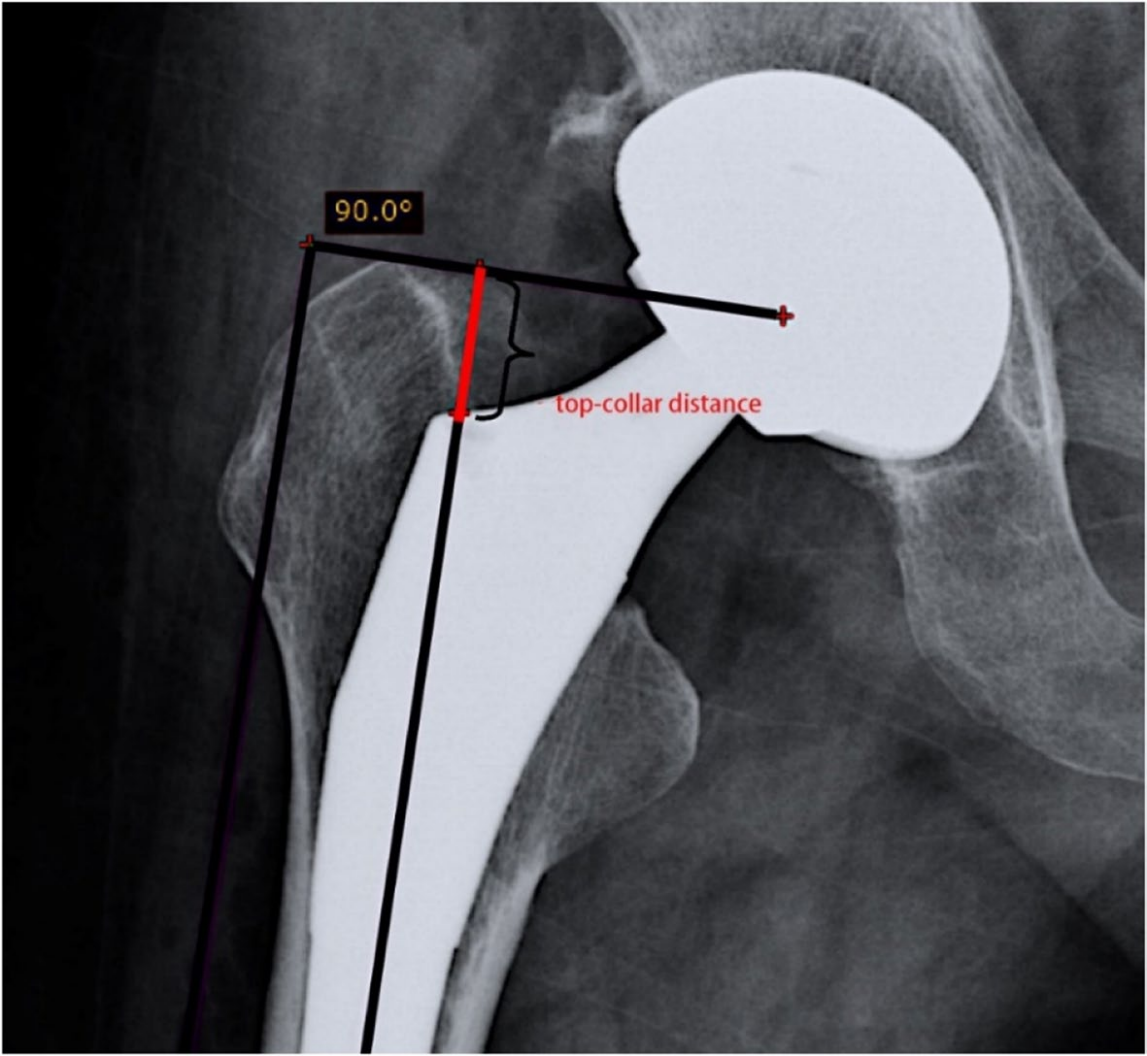
The top-collar distance indicates the vertical distance between the apex of the greater trochanter and the lateral collar of the femoral stem prosthesis

RadiAnt DICOM Viewer, as a medical measurement software, was used to measure the vertical distance (also known as top-collar distance) between the apex of the greater trochanter and the lateral collar of the femoral stem prosthesis on the standard pelvic X-ray in the anteroposterior position (Details see in Fig. 1). The average value was taken after three measurements. The top-collar distance at follow-up minus the top-collar distance immediately after surgery was the sinking distance at follow-up. The top-collar distances were measured at immediately, 3 months, 6 months and 1 year after operation, and the prosthesis sinking distances were calculated at 3 months, 6 months and 1 year after operation.

### Statistical analysis

SPSS 17.0 statistical software (USA) was used for statistical analysis. Measurement data, such as Harris score and prosthetic-sinking distance, were expressed as mean ± standard deviation (Mean ± SD). Independent sample t test was used for comparison between two groups, and paired sample t test was used for comparison in groups at different times. Chi-square test was used for enumeration data, such as the incidence of postoperative complications. When *P* < 0.05 was considered statistically significant.

## Results

### Patient characteristics

**Fig. 2:**
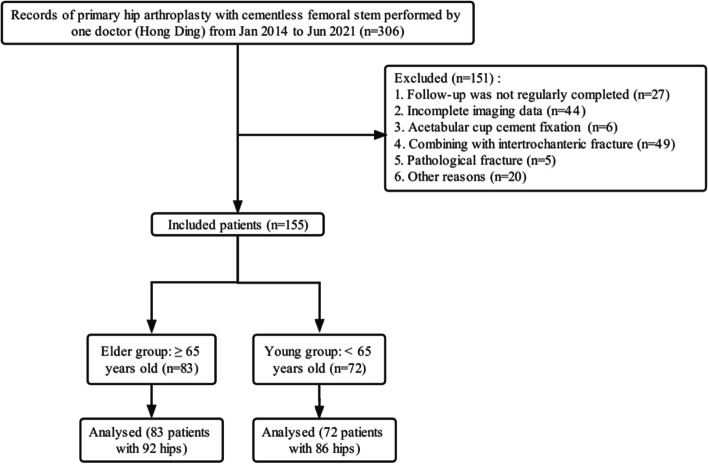
Flow chart of patients included in this study

As shown in Fig. 2, 306 patients with the primary hip arthroplasty of cementless femoral stem completed from January 2014 to June 2021 were reviewed. After excluding patients who did not meet the inclusion and exclusion criteria, only 155 patients with 178 hips were included in this study. In the young group (≤ 64 years old), there were 29 males and 43 females, a total of 72 patients with 86 hips. In the elder group (≥ 65 years old), there were 35 males and 48 females, a total of 83 patients with 92 hips. The disease types mainly included avascular necrosis of the femoral head in 61 hips, hip fractures in 69 hips, Posttraumatic arthritis in 16 subjects and so on. A total of 85 patients had Singh index ≤ 3, including 60 hips in the elder group and 25 hips in the young group. Detailed demographic data are shown in Table 1.

**Table 1:** Demographics of patients

	Elder group	Young group
Number of patients	83	72
Number of hips	92	86
Age(years)	78.9(65–98)	50.6(25–64)
Gender(Male/Female)	35/48	29/43
Side(left/right)	43/49	44/42
Body mass index(kg/m^2^)	25.83 ± 2.99	25.04 ± 2.88
Diagnoses		
Osteoarthritis	7	3
Posttraumatic arthritis	8	8
Fractures	51	18
Avascular necrosis	17	44
Rheumatoid arthritis	4	5
Sequela of developmental hip dysplasia	5	8
Singh index		
> 3	32	61
≤ 3	60	25
Surgery		
Total arthroplasty	57	77
Hemiarthroplasty	35	9

### Clinical results

**Fig. 3:**
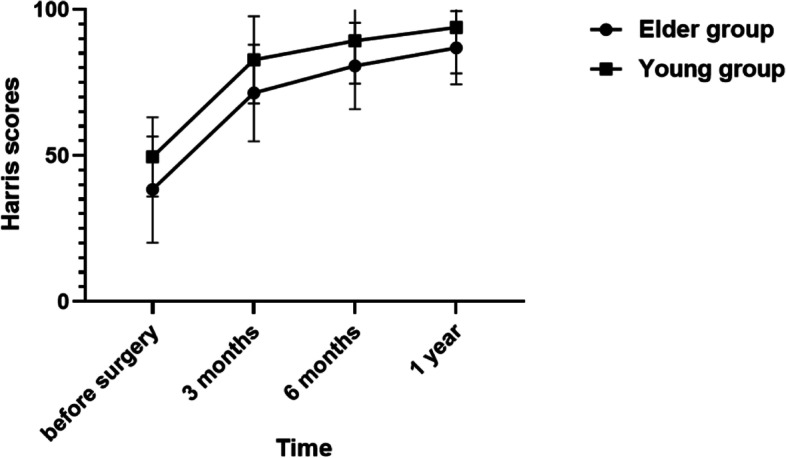
Harris scores at different follow-up time

The Harris score in the young group increased from 49.51 ± 13.59 before operation to 93.84 ± 15.75 at last follow-up (*P* < 0.01). The Harris score in the elder group increased from 38.35 ± 18.21 preoperatively to 86.91 ± 12.55 at last follow-up (*P* < 0.01). The details are shown in Fig. 3. It can be seen that both groups of patients achieved satisfactory functional results with cementless femoral stem prosthesis.

### Radiography

As shown in Table 2, the two groups were compared at the same time such as 3 months, 6 months, and 12 months after surgery, although the prosthesis distance of stem sinking was significantly higher in the elder group (all P < 0.05). In the elder group, even though the sinking distance in 6 months was significantly different from that in 3 months (2.44 ± 0.49 mm vs 2.10 ± 0.65 mm, *P* = 0.004), there was no significant difference between 12 and 6 months (2.53 ± 0.53 mm vs 2.44 ± 0.49 mm, *P* = 0.40). In the young group, there were no statistical differences between 6 and 3 months, or between 12 and 6 months.

**Table 2:** Radiological outcomes at different follow-up time points between groups

Outcomes	Elder group	Young group	*P* value
distance of stem sinking (mm)			
3 months	2.10 ± 0.65	1.75 ± 0.41	0.002
6 months	2.44 ± 0.49^a^	1.84 ± 0.43^c^	< 0.001
12 months	2.53 ± 0.53^b^	1.96 ± 0.39^d^	< 0.001

Abbreviation: ^a^ represents *P* = 0.004 compared with 3 months in elder group. ^b^ represents *P* = 0.40 compared with 12 months in elder group. c represents *P* = 0.29 compared with 3 months in young group. d represents *P* = 0.15 compared with 6 months in young group

### Complications

By the end of follow-up, ten patients in the elder group and six patients in the young group had a complication event (*P* = 0.36). No aseptic loosening of femoral stem prosthesis occurred in any of the patients. However, during the follow-up period, three patient in the younger group had mild thigh (1–3 points of VAS scores) pain within 6 months after surgery, which gradually eased. Three patients in the elder group had mild to moderate thigh pain after surgery (3–4 points of VAS scores), and both patients were relieved. One patient in the elder group suffered a periprosthetic fracture due to a fall after surgery and was diagnosed as Vancouver B1 fracture. The fracture healed after symptomatic treatment such as braking and brace protection. Hip dislocation occurred in two patient in the young group. Two patients in the elder group developed deep vein thrombosis, even with standardized anticoagulation and encouragement of early walk when get out of bed. After operation, the patient felt shorter lower limbs (> 1.5 cm), there were three patients in the elder group and one patient in the young group. None of the other patients had complications such as heterotopic ossification or deep infection (Table 3).

**Table 3:** Early and late complications

Complications	Elder group	Young group
Prosthesis loosening	0	0
Thigh pain	3	3
Periprosthetic fracture	1	0
Dislocation	0	2
Heterotopic ossification	0	0
Deep infection	0	0
Deep Venous Thrombosis	2	0
Lower limb shortening (> 1.5 cm)	4	1
Total	10	6

## Discussion

For patients with displaced femoral neck fracture of the hip and ischemic necrosis of the femoral head, total hip arthroplasty(THA) or hemiarthroplasty(HA) is often chosen to improve the quality of life and reduce bed-related complications, and its good clinical efficacy helps patients return to work and life soon [13]. At present, cemented fixation and cementless fixation are commonly used in femoral prosthesis fixation. The choice of cemented fixation for hip arthroplasty in elderly patients is easier to achieve initial stability and reduce the probability of intraoperative fracture, but it is also accompanied by some serious complications related to bone cement [4]. The cementless prosthesis is initially applied to young patients, because the biotype prosthesis takes advantage of bone growing into the pore of the prosthesis, and the hydroxyapatite coating on the prosthesis surface can be more closely combined with the femoral cortex of the patient, so it has a lower prosthesis revision rate [14]. Some researchers have begun to explore whether cementless prostheses can provide the same advantage in elderly patients. The use of cementless prosthesis can significantly reduce the operation time and avoid BCIS, and improve the perioperative survival rate of elderly patients.

Koettnitz et al. [15] proved that, compared with patients < 60 years, cementless femoral prosthesis implantation in a patient more than 80 years is a safe procedure with no increased risk of surgery-related complications. Huang et al. [7] also showed that the use of cementless THA can bring good clinical results for patients older than 80 years, but the deficiency is that fewer patients are included, and the evaluation results are only Harris score and postoperative complications, without imaging measurement results. But some scholars put forward the opposite view. Because elderly patients are often complicated with hip osteoporosis, they are considered as the preferred choice for femoral cemented fixation [16]. The use of cementless fixation may increase the risk of reoperation due to periprosthetic fractures [17]. Through a meta-analysis, Raja et al. [18] proved that cemented prosthesis group had better results and fewer complications, such as lower revision rate of prosthesis, and elderly patients should be given priority to use cemented prosthesis. Fernandez et al. [19] also demonstrated that in patients over 60 years with hip fractures, cemented hemiarthroplasty resulted in a significantly improved quality of life and a lower risk of periprosthetic fractures compared with cementless hemiarthroplasty. Therefore, it is still inconclusive whether cementless prosthesis should be used. Especially with the increasing elderly population, it is necessary to determine the exact efficacy of cementless prostheses. Current studies have focused on complications and functional outcomes without imaging evaluation. There was also a lack of results compared with younger patients. Therefore, we investigated whether the use of cementless femoral stem prostheses in older patients could provide similar clinical outcomes to those obtained in younger patients.

In this study, we found that in elderly patients with hip arthroplasty, the use of cementless prostheses on the femoral side can achieve similar results compared with younger patients. At 1-year follow-up, the Harris score in the elder group increased from 38.35 ± 18.21 to 86.91 ± 12.55 (*P* < 0.01). In addition, the recovery trend of the elder group was very similar to that of the young group, and the functional score increased significantly in the first three months, indicating that the cementless prosthesis can also provide stable fixation for elderly patients to get out of bed early, and enable patients to participate in functional training and return to life early. At the same time, this study found that within one year after surgery, the prosthesis in both groups had a certain degree of subsidence with the prolongation of time (see Table 1). At the same time, the distance of stem sinking in the elder group was larger than that of the young group at each follow-up time point, which may be related to the fact that most patients in the elder group had osteoporosis, resulting in slower binding between bone and prosthesis. The distance of stem sinking of the elder group in 6 months was more significant than that in 3 months (*P* < 0.05), but there was no significant difference in the distance of stem sinking between 12 and 6 months (*P* = 0.40), indicating that the cementless prosthesis began to become stable in 6 months. There was no significant difference in complication rates, and periprosthetic fractures were no more common in the older group than in the younger group.

As the first study to compare cementless femoral stem prostheses in older and younger patients. Short-term follow-up of functional outcomes, imaging findings, and complications was performed. It is found that elderly patients with cementless femoral stem prosthesis can quickly acquire good function outcome. Even though it takes longer for the implant to firmly bind to the cortical bone than in younger patients, the implant interface tends to stabilize at 6 months. There were no significant increases in incidence rate of periprosthetic fractures, infections, and deep vein thrombosis compared with younger patients. This study is similar to the results of Huang et al. [7] and Koettnitz et al. [15], and provides valuable imaging evidence on this basis.

The cementless femoral stem is mechanically pressed to achieve initial stability, and then achieve long-term bone ingrowth at the metal interface. Imaging results are very important indicator to estimate the initial stability of femoral stem prosthesis. The most intuitive expression of early stability on X-ray film is the relative displacement of prosthesis and medullary cavity. After comparing various measurement methods, Walker et al. [20] believed that it was highly reliable and repeatable to measure and evaluate the subsidence displacement of the prosthesis by taking the vertical distance from the vertex of the greater trochanter to the lateral collar of the prosthesis neck. Our results showed that the distance of stem sinking in the elder group was 2.10 mm in 3 months, which was similar to the 2 mm of Floerkemeier et al. [21]. This distance is similar to the sinking distance in fracture of Vancouver B2 with cementless hip arthroplasty [22] and less than the 4 mm of Seral et al. [23]. This indicates that the sinking distance achieved osseointegration within a safe range. However, compared with the 1.96 mm of young patients, the sinking distance (2.53 mm) in the elder group is still large. Considering that elderly patients are more complicated with osteoporosis, the bone metabolism rate is slowed down, and the osteogenic effect is weakened, so the osseointegration is longer and the sinking distance is relatively larger than that of young patients. To increase the contact area to achieve better compression matching, the medullary cavity file was used to expand the medullary cavity. The destruction of local intramedullary bone and blood supply by the medullary reaming itself may also be one of the factors affecting the early subsidence of the prosthesis.

However, osseointegration of femoral stem prosthesis was also achieved in the elder group within one year after operation. However, the initial stability of different types of prostheses may be determined by one or more factors, which is a comprehensive and complex process. The geometric shape, length, surface roughness and surface coating properties of femoral stem prosthesis, proximal shape of femoral bone marrow cavity and bone quality are all important factors affecting the initial stability of the prosthesis [24]. This study also suggests that osteoporosis may not be an absolute contraindication to the use of cementless femoral stems. With the progress and development of surgical technique, material science, biomechanics and other aspects, cementless femoral stem can also achieve good results in elderly patients. However, the cementless prosthesis may cause uneven pressure, lead to stress shieding, and cause thigh pain, which should also attract scholars' attention [25]. At present, there is no clear value of the safe distance of stem sinking, and we hope that our data and results can provide a new reference.

Meantime, the study still had some shortcomings. First, the follow-up period was relatively short, long-term survival rate and clinical function of the prosthesis still need further follow-up. Secondly, this study was a retrospective study, there was a high rate of loss to follow-up in the process of data collection, which may affect the study results. Thirdly, imaging data of the acetabular cup in total hip arthroplasty were ignored. Finally, subgroup comparisons of different bone qualities were not performed due to the limited number of patients.

## Conclusions

Older patients with cementless femoral stems have a slightly longer time to achieve metal-bone integration than younger patients, but short-term clinical outcomes can be restored to a satisfactory level with high safety. However, a multicenter prospective follow-up study with larger sample size and longer duration is still needed in the future.

## Data Availability

The original datasets are available from the corresponding author on reasonable request.
